# The Desmosomal Armadillo Protein Plakoglobin Regulates Prostate Cancer Cell Adhesion and Motility through Vitronectin-Dependent Src Signaling

**DOI:** 10.1371/journal.pone.0042132

**Published:** 2012-07-30

**Authors:** Carrie A. Franzen, Viktor Todorović, Bhushan V. Desai, Salida Mirzoeva, Ximing J. Yang, Kathleen J. Green, Jill C. Pelling

**Affiliations:** Department of Pathology, Northwestern University, Chicago, Illinois, United States of America; University of Kentucky College of Medicine, United States of America

## Abstract

Plakoglobin (PG) is an armadillo protein that associates with both classic and desmosomal cadherins, but is primarily concentrated in mature desmosomes in epithelia. While reduced levels of PG have been reported in localized and hormone refractory prostate tumors, the functional significance of these changes is unknown. Here we report that PG expression is reduced in samples of a prostate tumor tissue array and inversely correlated with advancing tumor potential in 7 PCa cell lines. Ectopically expressed PG enhanced intercellular adhesive strength, and attenuated the motility and invasion of aggressive cell lines, whereas silencing PG in less tumorigenic cells had the opposite effect. PG also regulated cell-substrate adhesion and motility through extracellular matrix (ECM)-dependent inhibition of Src kinase, suggesting that PG’s effects were not due solely to increased intercellular adhesion. PG silencing resulted in elevated levels of the ECM protein vitronectin (VN), and exposing PG-expressing cells to VN induced Src activity. Furthermore, increased VN levels and Src activation correlated with diminished expression of PG in patient tissues. Thus, PG may inhibit Src by keeping VN low. Our results suggest that loss of intercellular adhesion due to reduced PG expression might be exacerbated by activation of Src through a PG-dependent mechanism. Furthermore, PG down-regulation during PCa progression could contribute to the known VN-dependent promotion of PCa invasion and metastasis, demonstrating a novel functional interaction between desmosomal cell-cell adhesion and cell-substrate adhesion signaling axes in prostate cancer.

## Introduction

Prostate cancer (PCa) is the second leading cause of cancer mortality in the US [1], due mainly to the metastatic form of the disease [2]. The initial stages of tumor cell metastasis involve modulation of cell-cell and cell-substrate adhesive properties to facilitate cell detachment from the tissue of origin and invasion of local and distal tissues [3]. The down-regulation of cell-cell adhesion molecules is one of the major steps in the process of local and distal prostate tumor invasion. In particular, changes in the expression and function of cadherins and their associated proteins have gained attention as critical regulators of tumor progression [4], [5]. Loss of the adherens junction molecule, P-cadherin, is an early event in most prostate cancers [6], [7], while reduced expression of E-cadherin is connected with a more advanced and aggressive form of disease [8]. However, in spite of their expression in prostate tissues [9]–[11], little is known about the role of desmosomal cadherins in the initiation and progression of PCa.

Alterations in expression of cadherin-associated proteins in the armadillo family have also been linked to tumor progression and/or suppression [12]–[14]. One such protein is a close relative of β-catenin called plakoglobin (PG, also known as γ-catenin), which can bind to both classic and desmosomal cadherins, but is found primarily in desmosomes and is critically important in the morphogenesis of the skin and heart [10]. PG has been reported as a suppressor of tumorigenesis in cervical [15], bladder [16] and breast cancer [17], and clinical data indicate that PG is down-regulated in localized and most hormone refractory tumors in prostate cancer patients [18]. Interestingly, in some hormone refractory tumors, a mutated version of PG has been observed in nuclei correlating with increased Bcl2 expression and increased cell survival [18]. Whether these observed changes in PG play a causal role in PCa progression has not been determined.

In previous work, we showed that PG inhibits keratinocyte motility, in part through regulation of cell-cell and cell-substrate adhesion component expression [19], [20]. Through analysis of PCa patient tissue samples and a series of in vitro gain-and-loss of function studies, our data suggest a model by which PG regulates both cell-substrate interactions and cell-cell adhesion by attenuating vitronectin (VN)-dependent activation of Src. This is the first report that desmosomal molecules can regulate prostate cancer interactions with the extracellular microenvironment. Furthermore, our data raise the possibility that PG down-regulation during PCa progression could contribute to the known VN-dependent promotion of PCa invasion and metastasis to bone [21].

## Materials and Methods

### Cell Lines and Culture

LNCaP cells are functionally differentiated, androgen-sensitive human prostate adenocarcinoma cells derived from a lymph node metastasis [22]. C4-2B cells are an androgen-independent, bone metastatic derivative of LNCaP cells [23]. DU145 cells are moderately differentiated, androgen-inedpendent human prostate adenocarcinoma cells derived from a brain metastasis [24]. PC-3 cells are poorly differentiated, androgen-independent human prostate adenocarcinoma cells derived from a vertebral metastasis [25]. PC3-M is a highly metastatic variant of the PC-3 cell line [26]. ARCaP cells are androgen-repressed cells which express functional androgen receptor [27]. ARCaP_E_ cells are an epithelial variant of the parental ARCaP cells with low tumorigenicity and ARCaP_M_ cells are a highly metastatic mesenchymal variant of the parental ARCaP cells [28]. PC3-M cells, LNCaP cells, PC-3 cells (all gifts from Dr. Raymond C. Bergan, Northwestern University, Chicago, IL), DU145 cells (a gift from Dr. Lester F. Lau, University of Illinois, Chicago, IL) and C4-2B cells (a gift from Dr. R.S. Taichman, University of Michigan, Ann Arbor, MI) were cultured in RPMI 1640 medium (Mediatech) containing 10% heat-inactivated fetal bovine serum (FBS) (Invitrogen, Carlsbad, CA), 100 units/ml penicillin, and 100 µg/ml streptomycin (Mediatech). ARCaP_E_ and ARCaP_M_ cells (Novicure) were cultured in MCaP media (Novicure) containing 5% heat-inactivated FBS, 100 units/ml penicillin, and 100 µg/ml streptomycin (Mediatech).

### Reagents and Antibodies

pSrc (Y416) polyclonal antibodies and Src monoclonal antibodies (for western blotting and immunofluorescence studies in cell lines) were from Cell Signaling Technologies (Danvers, MA). pSrc polyclonal antibodies (for TMA staining), Collagen I polyclonal antibodies were from AbCam (Cambridge, MA). PG chicken polyclonal antibodies were from Aves Labs (Eugene, OR). GAPDH monoclonal antibody was from Millipore (Temecula, CA). HRP-conjugated goat anti-mouse and goat anti-rabbit secondary antibodies were from Bio-Rad Laboratories (Hercules, CA). HRP-conjugated goat anti-chicken secondary antibody was from Kirkegaard & Perry Laboratories (Gaithersburg, MD). Vitronectin (VN) monoclonal antibody, fibronectin polyclonal antibodies, laminin monoclonal ascites fluid, and VN from human plasma were from Sigma. PP2 selective Src inhibitor was from Calbiochem (San Diego, CA). Dispase II enzyme was obtained from Roche (Basel, Switzerland). PG siRNA pool and control siRNA #2 non-targeting sequence were obtained from Dharmacon (Lafayette, CO). caSrc adenovirus was a generous gift from Dr. Paul L. Stein (Northwestern University). Diff-quik Stain set was from Dade Behring.

### Adenoviral Constructs and Transduction

The pAdEasy adenovirus packaging system, kindly provided by Dr. Warren G. Tourtellote (Northwestern University Feinberg School of Medicine) was used to generate previously characterized myc-tagged, full-length human PG [29] according to published protocols [30]. Cell infection rates were monitored using GFP expression in tandem with PG; at least 30% of re-plated cells still retained GFP expression.

### siRNA Transfections

ARCaP_E_, LNCaP, and C4-2B cells were grown to 30% confluency, and then were transfected with either individual or a pool of 4 small interfering ON-TARGETplus RNA (siRNA) for human PG or with #2 control non-targeting sequence (Dharmacon, Lafayette, CO) at the final concentration of 20 nM using Dharmafect 1 transfection reagent (Dharmacon) according to manufacturer’s recommendations. PG siRNA sequences used were as follows: 5′-AGACAUACACCUACGACUC-3′; 5′-UGAGUGUGGAUGACGUCAA-3′; 5′-CCACCAACCUGCAGCGACU; 5′-UGUACUCGUCGGUGGAGAA-3′.

### Scratch Wound Assay

PC3-M and ARCaP_M_ cells were plated in 24-well plates and allowed to attach and spread. Cells were transduced with GFP- or PG-containing adenovirus. At 24 hours after transduction, a scratch wound was made by scratching the confluent monolayer with a 20 µl pipette tip. For Src inhibition studies, cells were treated with medium containing the Src inhibitor PP2 or DMSO solvent control for 24 hours prior to the scratch wound being made. Cells were imaged immediately after wounding and 24 hours after wounding. At 24 hours after wounding, cells were lysed with Urea Sample Buffer (USB) and lysates were used to test for expression of PG. ARCaP_E_ and C4-2B cells were plated in 24-well plates and allowed to attach and spread. Cells were transfected with control or PG siRNA. A scratch wound was made 96 hours after transfection. For Src activation studies, ARCaP_E_ cells were transduced with GFP-adenovirus or constitutively active Src (caSrc)-adenovirus for 24 hours prior to the scratch wound being made. Cells were imaged immediately after wounding and 18 hours (C4-2B cells) or 24 hours (ARCaP_E_ cells) after wounding. At 24 hours after wounding, cells were lysed with USB and lysates were analyzed by western blotting to test for expression of PG. The percentage of wound closure was determined using ImageJ software.

### Matrigel Invasion Assay

The Matrigel invasion assay was performed as previously described [31] with the following modifications. The inner well of transwell inserts (0.8 µm; BD Biosciences) were coated with 100 µl of Matrigel Basement Membrane Matrix (BD Biosciences) and allowed to permeabilize at room temperature for 1 h. The remaining liquid was removed, and filters were washed with serum-free medium and allowed to air dry. PCa cells were detached according to cell line specific protocols (ARCaP_E_, ARCaP_M_, C4-2B, DU145, and PC-3 cells were detached using trypsin, while LNCaP and PC3-M cells were detached using calcium sequestration) and washed with serum-free medium, and 5×10^5^ cells were added to the inner invasion chamber. Cells were allowed to invade for 24 h; non-invading cells were removed from inner wells using a cotton swab, and invading cells adherent to the bottom of the membrane were fixed and stained using the Diff-Quik staining kit (DADE AG). The invading cells were enumerated by upright stereo microscope using a 10x objective (Nikon).

### Immunoblotting

ARCaP_M_, C4-2B, and PC3-M cells were plated in 6-well plates and allowed to attach and spread. Cells were transduced with GFP-containing adenovirus or PG-containing adenovirus, harvested 24 hours later with USB and subjected to SDS-PAGE. After transfer to a nitrocellulose membrane, immunoblot analysis was performed with antibodies against PG, pSrc, Src, LN, FN, VN, and GAPDH. ARCaP_E_ and C4-2B cells were plated in 6-well plates and allowed to attach and spread. Cells were transfected with PG siRNA or #2 control sequence siRNA. After 96 hours, cells were lysed with USB and subjected to SDS-PAGE, transferred to a nitrocellulose membrane, and then subjected to immunoblot analysis with antibodies against PG, VN, FN, Col IV, and GAPDH. Quantitations of band intensities were carried out using Analyze Gels tool of the ImageJ software.

### Mechanical Strength (Dispase) Assay

LNCaP, C4-2B, and PC3-M cells were plated in 6-well plates, and allowed to attach and spread. Cultures were transduced with GFP-containing adenovirus or PG-containing adenovirus, and after 24 hours the dispase assay was performed. For Src inhibition studies, cells were treated with medium containing PP2 (10 µM) or DMSO (solvent control) for 24 hours prior to performing the dispase assay as described [32]. Briefly, confluent cell cultures were washed with PBS and incubated with 2.4 U/ml dispase for 30 min at 37°C. Released monolayers were subjected to mild mechanical stress, and then fixed with formalin. Fragments were counted with a dissecting scope (Leica, MZ6) as described [32] or imaged with a Hamamatsu Orca digital camera (model C4742-95) and analyzed using MetaVue imaging software (Molecular Devices). Data are expressed as average number of fragments ± SEM. Statistical analysis was done using a Student’s *t* test.

### Hanging Drop Aggregation Assay

The hanging drop assay was performed as described previously [32], with the following modifications. Cultures of PCa cell lines were detached as described above, centrifuged, and resuspended in the appropriate culture medium. Drops (20-µl) of cell suspensions were seeded onto the inner surface of a 35-mm culture dish lid and cultured for 20 hours. To limit evaporation, 2 ml PBS was added to the bottom of the culture dishes. To compare similar densities of cells suspended in the drop, 4×10^3^ cells were examined. To examine the ability of cells to form aggregates after 24 hours, the culture dish lids were inverted, and the hanging drops were flattened with a glass coverslip for imaging. To examine the adhesive strength of the cellular aggregates, parallel cultures were triturated 10 times through a 20-µl pipette tip (pre-rinsed with 0.1% Triton X-100/PBS) then rinsed 3 times in PBS prior to imaging. Five random fields of phase-contrast images from each hanging drop were acquired using a Zeiss Axiovert 200 inverted microscope with a Zeiss Axiocam camera and Zeiss Axiovision software. The total number of cell clusters was counted from triplicate hanging drops.

### Immunofluorescence

ARCaP_E_, ARCaP_M_, LNCaP, C4-2B, DU145, PC-3, and PC3-M cells were grown on glass coverslips, rinsed in phosphate-buffered saline (PBS) and fixed in anhydrous methanol for 2 min at −20°C. They were then incubated with chicken anti-PG (1407) (1∶1000) and mouse anti-E-cadherin (HECD-1) antibodies (1∶100) overnight at 4°C. Primary antibodies were visualized using Alexa Fluor 350-, 488- and 568-conjugated goat secondary antibodies against mouse, rabbit and chicken IgG (1∶400), respectively. Coverslips were examined with a Leica upright microscope model DMR (Deerfield, IL), and images were captured using a Hamamatsu Orca digital camera model C4742-95 (Bridgewater, NJ) and Metamorph 7.6 (Molecular Devices) imaging software.

### Tissue Microarray Staining, Imaging, and Fluorescent Intensity Analysis

The paraffin-embedded prostate tissue microarray (BC19021) containing 72 cores from 20 cases was purchased from US Biomax (Rockville, MD), and was processed for immunohistochemical staining as described [33]. Antigen retrieval was performed by heating the glass slide to 95°C in 0.01 M citrate buffer. The microarray was blocked in 2% normal goat serum (Jackson ImmunoResearch Laboratories) and 1% BSA for 60 min at 37°C, then incubated with primary antibodies against PG, VN and pSrc overnight at 4°C in a humidified chamber. The microarray was incubated in AlexaFluor-conjugated secondary anti-chicken (633), mouse (488) and rabbit (568) antibodies for 60 min at 37°C, and the coverslip was mounted in polyvinyl alcohol. PG, VN, and pSrc were visualized using confocal microscopy (Cell Imaging Facility, Northwestern University), within 3 days of processing. Identical acquisition settings were used for all images to ensure that image pixel intensity can be compared. Images were taken at 40X magnification, with at least 4 images per tissue core taken. Relative fluorescent intensities were determined by using MetaMorph 7.6 software (Molecular Devices) as described before [34] with the following modifications. Each raw, unsaturated image was set to a 16 bit range (scale of 0 for low and 255 for high) and was inclusively thresholded. The Measure Grid function was used to select regions of the tumor to be analyzed. Each grid was 40×40 squares (30.86 µm^2^/square). The average intensity of the thresholded areas was automatically calculated by MetaMorph for each grid. Grid squares with intensity of 0 were excluded from the average intensity calculation for the whole grid. Tumor staging has been characterized by U.S. Biomax, according to the TNM scale used by UICC [35]. Primary tumor (T) staging criteria for prostate cancer was used as follows: T1, Clinically unapparent tumor; T2, Tumor confined within prostate; T3, Tumor extends through the prostate capsule; T4, Tumor invades adjacent structures other than seminal vesicles. Detailed description of tissues used for the microarray may be found on the company website http://www.biomax.us/tissue-arrays/Prostate/BC19021. In addition, prostatic adenocarcinoma was graded by an expert genitourinary pathologist (Ximing J. Yang) using the Gleason grading system, which is solely based on the architectural features of the tumor cells.

### Statistical Methods

Cell culture experiments were carried out with 3 or more replicates. Values are given as mean with error bars indicating SEM. Statistical analyses were conducted using GraphPad Prism software version 5.02 for Windows (San Diego, USA). All statistical tests were 2-sided. Student’s t-test for bar graph comparisons was performed. Linear regression was performed for line graphs comparing expression of VN and PG or pSrc and PG, with the R squared value representing the goodness of the fit of the line. p<0.1 was considered statistically significant.

## Results

### PG is Mislocalized and its Expression is Reduced in Prostate Cancer Tissue Samples and Cell Lines

**Figure 1:**
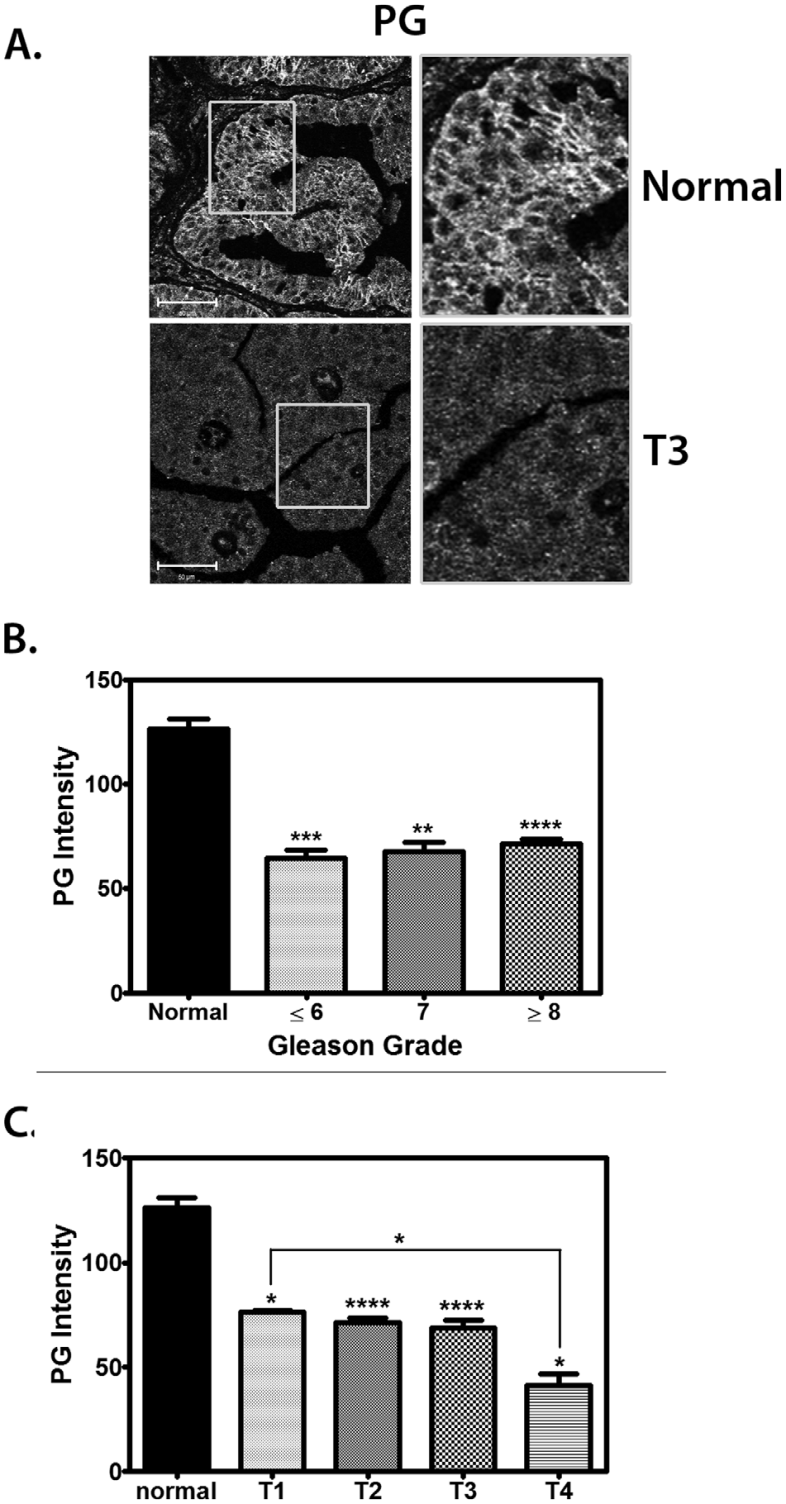
PG is down-regulated in invasive prostate cancer. A–C. A prostate tissue microarray was stained with an antibody against PG. A, **Representative immunofluorescence** image showing PG expression in normal prostate tissue (top) and T3 (bottom) prostate tissue. Bar represents 50 µm. B. Quantification of PG expression in normal prostate tissue as compared to tissue from prostate tumors with Gleason Grade less than or equal to 6 (6 tumors), 7 (5), or greater than or equal to 8 (33). PG expression is high in normal tissue (black) and is decreased by ∼50% in tumor tissue of all Gleason grades. Gleason Grading was performed by surgical pathologist Ximing Yang. C. Quantification of PG expression in normal prostate tissue as compared to stage T prostate tumor tissue. PG is highly expressed in normal tissue (black) and expression is diminished by ∼40% in stage T1–T3 prostate tumor tissue and by almost 70% in T4 prostate tumor tissue. Tumor staging has been characterized according to the TNM scale used by UICC [35]. Primary tumor (T) staging criteria for prostate cancer was used as follows: T1, Clinically unapparent tumor (3 tumors); T2, Tumor confined within prostate (33); T3, Tumor extends through the prostate capsule (9); T4, Tumor invades adjacent structures other than seminal vesicles (3). In addition, there were 8 normal prostate tissue samples in the array. Graphs represent averages +/− SEM. *P<0.05; **P<0.002; ***P<0.0005; ****P<0.0001, by paired t test.

Several reports in the literature have suggested that PG expression is reduced in clinical PCa samples compared to normal tissue [18], [36], [37]. However, whether modulation of PG expression is responsible for any of the physiological and molecular events in PCa tumorigenesis has not yet been mechanistically studied. To address this issue and gain a better understanding of the relationship between PG expression and the extent of primary prostate tumor invasion, a PCa tissue microarray with 72 cores from 20 prostate cancer patients was analyzed for PG expression. We observed a decrease in overall PG expression in all malignant samples compared to normal tissue, as well as a disappearance of PG from the cell-cell borders where it is normally localized (Fig. 1A). Analysis of the PG fluorescence intensity revealed, on average, ∼50% less PG in primary tumor samples of all Gleason Grades and staged T1 through T3, compared with normal tissue (Fig. 1B–C). Moreover, in invasive T4 tumors there was an additional ∼30% decrease in PG (Fig. 1C). These findings indicated that PG expression was reduced and mislocalized in primary prostate cancer tissue compared to normal prostate tissue. In addition, further loss of PG in invasive (T4) tumor samples raises the possibility that PG plays a role in suppressing PCa invasion.

**Figure 2:**
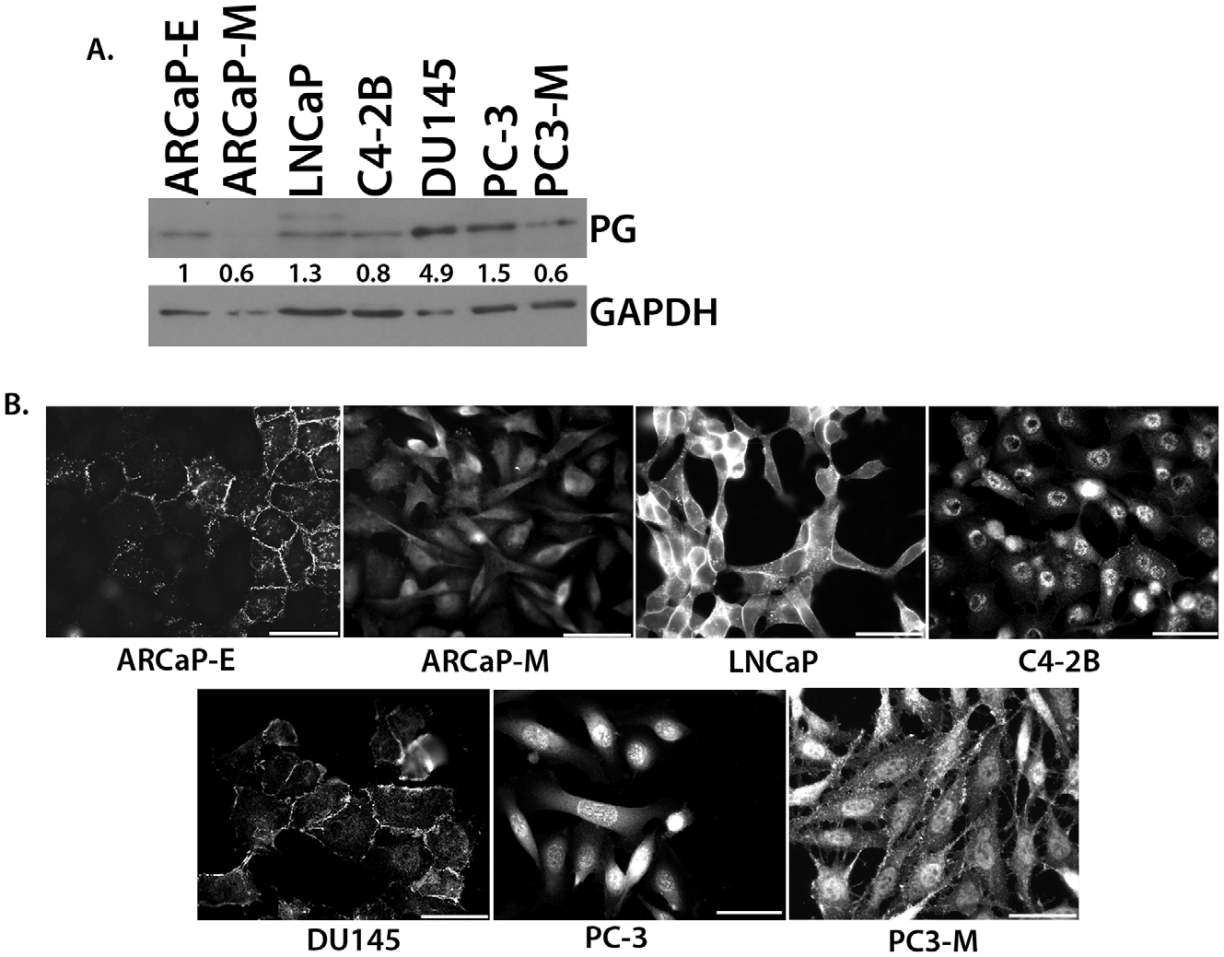
Expression levels and localization of PG in prostate cancer cell lines. A. Prostate cancer cell lines were lysed and subjected to SDS-PAGE, followed by immunoblotting with antibodies against PG and GAPDH. PG levels were determined by normalizing to GAPDH levels for each cell line using Image J. The immunoblot shows that the expression of PG in prostate cancer cell lines inversely correlates with the degree of aggressiveness of the cell line. B. Prostate cancer cell lines were plated onto coverslips and allowed to attach and spread. Immunofluorescence was performed using antibodies against PG to demonstrate the localization of PG in ARCaP_E_, ARCaP_M_, LNCaP, C4-2B, DU145, PC-3, and PC3-M cell lines. The immunofluorescence shows weaker staining and a loss of cell-cell border localization of PG in the more aggressive cell lines (ARCaP_M_, C4-2B, and PC3-M). Representatives of at least three independent immunoblots are shown, with numbers representing GAPDH normalized PG protein levels for the blot shown. All the PG protein levels were normalized to ARCaP_E_ levels within each blot. The average level and standard deviation of PG protein levels in panel A is as follows: ARCaP_E_ 1.0, ARCaP_M_ 0.3+/−0.2, LNCaP 1.5+/−0.5, C4-2B 0.5+/−0.2, DU145 3.4+/−1.2, PC-3 1.0+/−0.4, PC3-M 0.5+/−0.2.

Next, PG expression levels and distribution were assessed in a panel of seven PCa cell lines varying in their tumorigenic properties. In general, the cell lines described in the literature as having more metastatic potential (ARCaP_M_, C4-2B, and PC3-M) exhibited several-fold lower PG expression than did less aggressive lines (LNCaP and ARCaP_E_). While moderately differentiated DU145 cells expressed more PG than the other cell lines, PG exhibited a more diffuse cytoplasmic and nuclear localization rather than being concentrated at cell-cell interfaces (Fig. 2A, B). Similarly, the aggressive ARCaP_M_ and C4-2B lines exhibited dim and diffuse staining for PG. Lastly, the metastatic variant of the PC-3 cell line, PC3-M cells, expressed less PG than the PC-3 cells (Fig. 2A, B). Interestingly, even though they exhibited overall lower PG levels than PC3-M cells, C42B cells still show some nuclear staining (Fig. 2B). Overall these data suggest a trend of decreased PG expression and a loss of cell-cell border localization of PG in the aggressive PCa cell lines.

### PG Promotes Cell-cell Adhesion of PCa Cells

**Figure 3:**
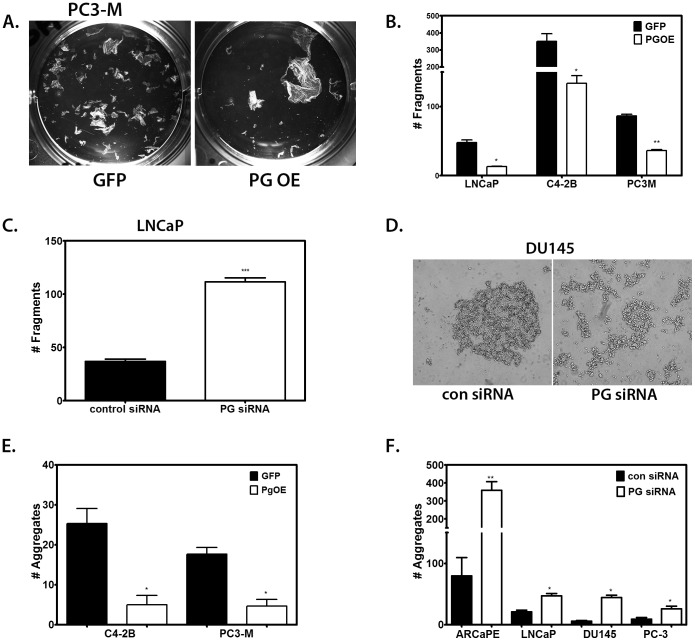
PG promotes cell-cell adhesion in prostate cancer cell lines. A. Representative image of a dispase assay in PC3-M cells after transduction with PG OE adenovirus or control GFP adenovirus. B. Quantitations of dispase assays performed in triplicate; LNCaP, C4-2B, or PC3-M cells were plated in 6-well plates, and allowed to attach and spread. Cultures were transduced with GFP-containing adenovirus (GFP) or PG-containing adenovirus (PG OE), and after 24 hours the dispase assay was performed. Overexpression of PG in all 3 cell lines strengthens cell-cell adhesion. C. LNCaP cells were plated in 6-well plates and allowed to attach and spread. The cells were then transfected with control siRNA or PG siRNA pool. The dispase assay was performed 96 hours after transfection. Suppression of PG expression results in a weakening of cell-cell adhesion in LNCaP cells. D. Representative image of a hanging drop assay in DU145 cells after transfection with control or PG siRNA pool. E–F. Quantitation of hanging drop assays performed in triplicate; C4-2B and PC3-M cells were transduced with GFP- or PG-containing adenoviruses (E), ARCaP E, LNCaP, DU145 and PC3 cells were transfected with control siRNA or PG siRNA pool (F). Aggregation assays were done 24 h (E), or 96 h (F) after the treatment. Graphs represent averages +/− SEM. *P<0.06; **P<0.005; ***P<0.0007, by paired Student t test.

In view of the fact that a hallmark of invasive cancer is the ability of malignant cells to coordinate the weakening of their cell-cell adhesions with changes in the functional interactions with the underlying substrate [3], we next investigated whether experimental manipulation of PG levels (Fig. S1 and S2) affected cell-cell adhesion in PCa cells. PC3-M cells, C4-2B cells, and LNCaP cells were transduced with PG-expressing adenovirus and a dispase assay was performed to measure intercellular adhesion strength (Fig. 3A). Cell-cell adhesion was significantly increased in all cells transduced with PG-expressing adenovirus (PG OE) compared to cells transduced with control (GFP) adenovirus (Fig. 3A–B).

Interestingly, overexpression of PG in LNCaP cells, which already express substantial levels of PG, led to a further increase in intercellular adhesion strength and cell-cell junction staining of E-cadherin (Fig. 3B and Fig. S3), suggesting that the presence of additional PG may further stabilize adherens junctions in PCa. Additionally, in ARCaP_M_ cells, overexpression of PG resulted in an up-regulation of the desmosomal component desmoplakin (Fig. S3), indicating a potential stabilizing effect of PG on desmosomal junctions in PCa as well. On the other hand, knocking down PG expression in LNCaP cells led to a substantial weakening of cell-cell adhesions (Fig. 3C). Moreover, down-regulation of PG led to a decrease of both E-cadherin and desmosomal cadherin desmoglein 2 in ARCaP_E_ cells (Fig. S3), highlighting again the importance of PG for the stability of adherens and desmosomal junctions in PCa. These results collectively support the notion that PG expression strengthens cell-cell adhesion in PCa cells.

To further confirm the effect of PG on PCa cell-cell adhesion, we performed aggregation (hanging drop) assays of cells in suspension (Fig. 3D). This assay was previously used to quantify the adhesion strength of a variety of epithelial cells, including A431, MDCK and SCC68 [38]–[40]. To test cell-cell adhesion strength we subjected aggregates to shear force. The number of fragments produced by shearing of aggregates was significantly decreased in the cell lines overexpressing PG compared to control (Fig. 3E). In addition, knocking-down PG led to a significant increase in the number of fragments, suggesting the weakening of cell-cell adhesion resistance to shear force (Fig. 3D, F).

### PG Inhibits Motility and Invasion in PCa Cell Lines

**Figure 4:**
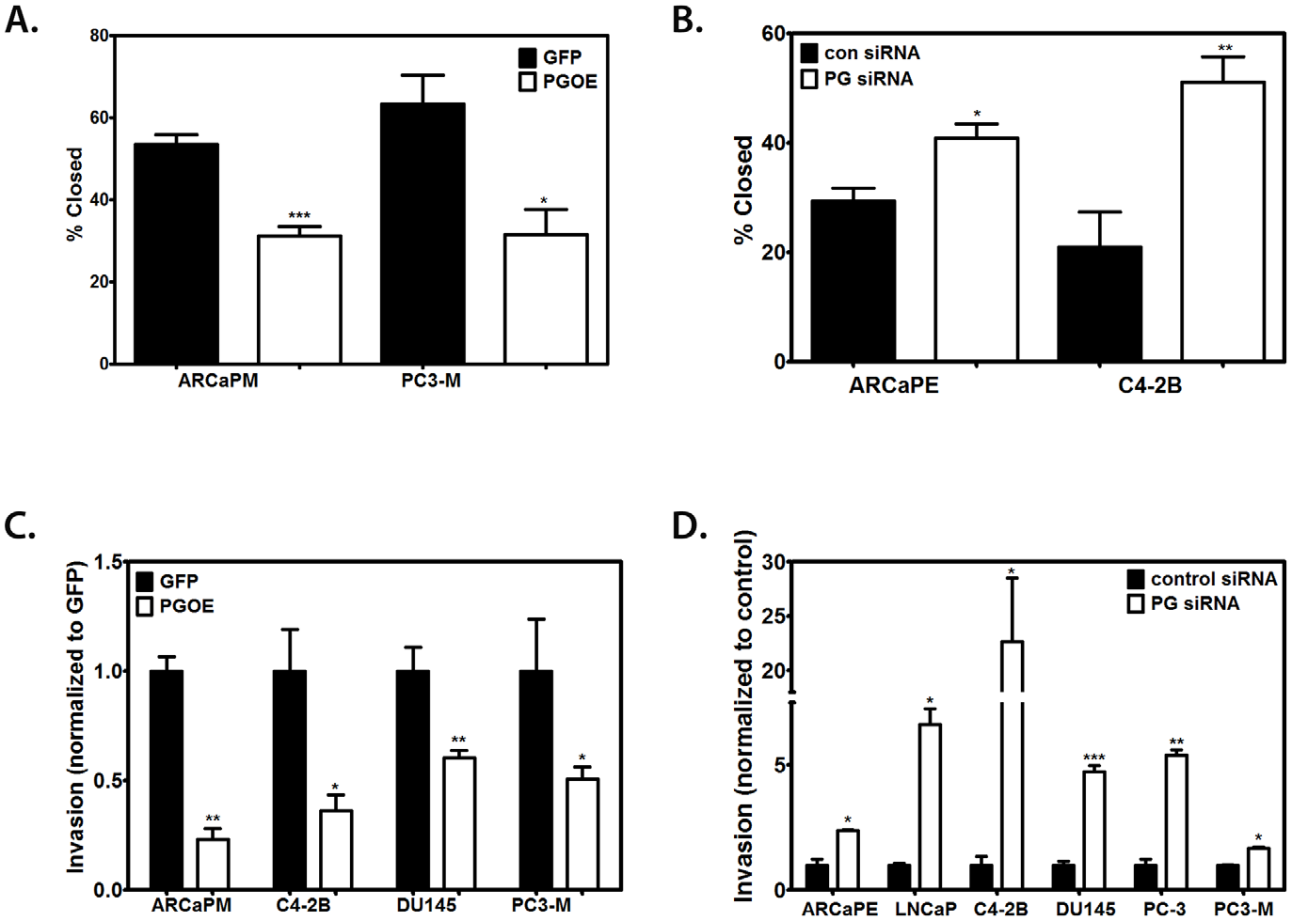
PG inhibits motility in prostate cancer cell lines. A. PC3-M and ARCaP_M_ were plated in 24-well plates and allowed to attach and spread. The cells were then transduced with GFP-containing adenovirus or PG-containing adenovirus, and after 24 hours a scratch wound was made. Images were taken at 0 and 24 hours and the % wound closure was calculated by comparing the size of the wound at 24 hours to the 0 hour time point for each condition. Overexpression of PG suppresses motility in these two cell lines. B. ARCaP_E_ and C4-2B cells were plated in 24-well plates and allowed to attach and spread. The cells were then transfected with control siRNA or PG siRNA pool, and 72 hours after transfection, the scratch wounds were made. Suppression of PG leads to an increase in motility in ARCaP_E_ and C4-2B cells. Scratch wound assay done in triplicate in prostate cancer cell lines after overexpression or knockdown of PG. C. ARCaP_M_, C4-2B, DU145 and PC3-M cells were transduced with GFP-containing adenovirus or PG-containing adenovirus, and after 24 hours plated onto Matrigel coated transwell membranes for an invasion assay. Overexpression of PG suppresses invasion in these four cell lines. D. ARCaPE, LNCaP, C4-2B, DU145, PC-3 and PC3-M cells were transfected with control siRNA or PG siRNA pool, and 72 hours after transfection, plated onto Matrigel coated transwell membranes for an invasion assay. Suppression of PG leads to an increase in invasion in these six cell lines. Invasion assays were done in triplicate. Average of the total number of invading cells is presented. Graphs represent averages +/− SEM. *P<0.1; **P<0.05; ***P<0.001, by paired Student t test.

As weakening of cell-cell adhesions has been reported to increase cell motility [41], we next investigated whether PG status in cells correlated with their relative motility. Towards this end, we performed a scratch wound assay to compare the motility of PC3-M, ARCaP_M_, ARCaP_E_, and C4-2B cells. The assay revealed that PC3-M and ARCaP_M_ cells were highly motile (with over 50% of the wound closed in 24 h), whereas ArCaP_E_ and C4-2B cells were less motile (around 20% closed in 24 h) (Fig. 4A, B). We then overexpressed PG in PC3-M and ARCaP_M_ cells, which decreased their motility by 2-fold compared with the control (Fig. 4A). At the same time, knockdown of PG in ARCaP_E_ and C4-2B cells resulted in an approximately 50% and over 2-fold increase in their motility, respectively (Fig. 4B). These findings collectively support the concept that ectopically expressing PG inhibits motility of PCa cells, while silencing PG increases their motility.

A regulatory role for PG in PCa cell motility suggested a possibility that PG may be responsible for inhibition of PCa cell invasion. To test this hypothesis we used a Matrigel invasion assay. To assess the potential role of PG in modulating PCa cell motility we performed both overexpression and silencing of PG in a number of PCa cell lines (Fig. 4C–D). Overexpression of PG decreased the invasiveness of PCa cells by 2- to 4-fold (Fig. 4C). Likewise, in all 6 cell lines in which we knocked PG down, a significant increase in cell invasiveness was observed. The increases ranged from 2-fold in PC3-M and ArCaP_E_ cells, to over 20-fold in C4-2B cell line (Fig. 4D). These results suggest that PG reduces the invasion potential of PCa cells.

### PG Regulates PCa Cell Motility and Cell-cell Adhesion through Inhibition of Src Kinase

**Figure 5:**
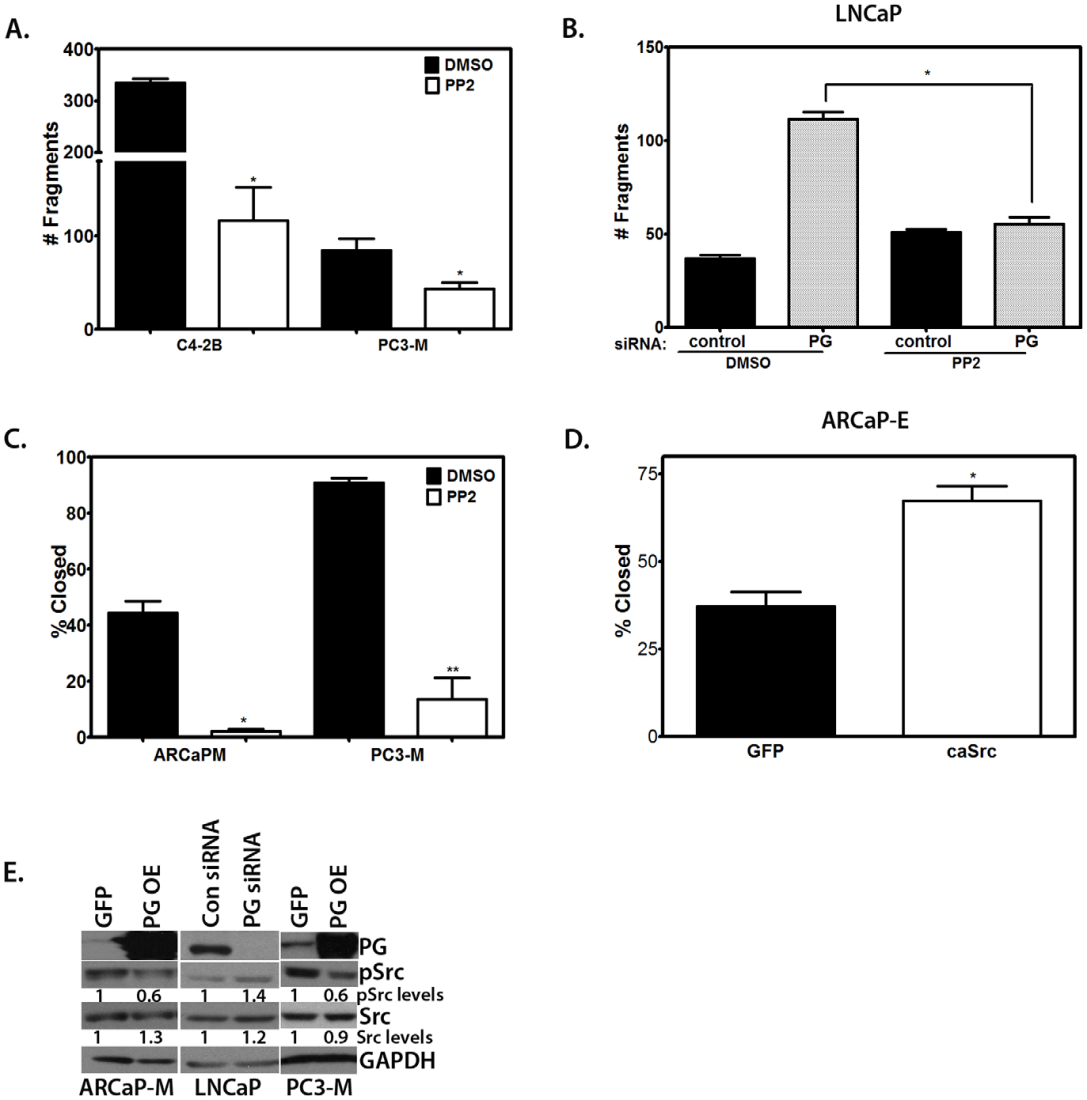
Src regulates motility and PG down-regulates Src activity in prostate cancer. A–B. PC3-M, C4-2B (A), and LNCaP cells (B) were plated in 6 well plates and allowed to attach and spread. LNCaP cells were transfected with control siRNA or PG siRNA (a pool of 4 sequences). PC3-M, C4-2B, and LNCaP cells were treated with medium containing PP2 (10 µM) or DMSO (solvent control) for 24 hours prior to performing the dispase assay. Inhibition of Src strengthens cell-cell adhesion in PCa cells in general and is able to rescue cell-cell adhesion in PG-deficient cells. C. PC3-M and ARCaP_M_ cells were plated in 24-well plates and allowed to attach and spread. The cells were treated with medium containing the selective Src- family kinase inhibitor PP2 (10 µM) or DMSO (solvent control) for 24 hours prior to performing the scratch wound assay. D. ARCaP_E_ cells were plated in 24-well plates and allowed to attach and spread. The cells were transduced with GFP-containing adenovirus or caSrc-containing adenovirus, and after 24 hours a scratch wound was made. Activity of Src is directly correlated with motility of PCa cells. E. ARCaP_M_, PC3-M, and LNCaP cells were plated in 6-well plates and allowed to attach and spread. LNCaP cells were transfected with control siRNA or PG siRNA pool and after 96 hours, the cells were lysed. ARCAP_M_ and PC3-M cells were transduced with GFP-containing adenovirus or PG-containing adenovirus, and after 24 hours the cells were lysed. The lysates were subjected to SDS-PAGE followed by immunoblotting with antibodies against PG, pSrc, Src, and GAPDH. Levels of pSrc were determined by normalizing to Src levels for each cell line using Image J. Phosphorylation (activation) of Src is inversely correlated with PG levels. Representatives of at least three independent immunoblots are shown, with numbers representing GAPDH normalized pSrc/Src ratio for the blot shown. The average ratio and standard deviation of pSrc/Src normalized for loading by GAPDH is as follows: ARCaP_M_ 0.6+/−0.2, LNCaP 1.7+/−0.2, PC3M 0.7+/−0.1. Graphs represent averages +/− SEM. *P<0.04; **P<003; ****P<0.0001, by paired Student t test.

Src kinase is one of the most important regulators of cell-cell adhesion and motility [42], and it has been implicated in PCa progression [43]. Since recent data suggest a functional interaction between PG and Src [19], [20], we investigated whether experimental modulation of Src activity in cells affected PCa cell-cell adhesion and cell motility. Our results demonstrate that inhibition of Src strengthened cell-cell adhesion in the PC3-M and C4-2B cell lines (Fig. 5A). To determine whether inhibiting Src activity would increase cell-cell adhesion in a PG-deficient background, we performed a dispase assay in LNCaP cells which had been transfected with siRNA to knockdown PG expression. Whereas PG ablation led to an almost 3-fold decrease in cell-cell adhesion strength compared to control cells, treatment with the Src-family kinase inhibitor PP2 completely abolished this effect of PG loss, restoring cell-cell adhesion to normal levels (Fig. 5B). These results support our hypothesis that Src activity is required for PG-dependent effects on cell-cell adhesion.

In parallel studies, we inhibited or activated Src, then employed the scratch wound assay to assess cell motility. Treatment of ARCaP_M_ and PC3-M cells with the selective Src-family kinase inhibitor PP2 led to a dramatic decrease in cell motility (Fig. 5C). Conversely, transduction of ARCaP_E_ cells with a caSrc-expressing adenovirus resulted in a nearly 2-fold increase in cell motility compared to cells transduced with GFP-adenovirus (Fig. 5D). These findings provide additional evidence that Src activity is required for weakening of cell-cell adhesion and induction of cell motility in PCa cell lines.

In order to test whether the level of PG expression affected the level of Src activity in PCa cells, we examined the levels of activated Src in prostate cancer cell lines after knockdown or overexpression of PG. When PG was overexpressed in ARCaP_M_ and PC3-M cells, the levels of pSrc were decreased. In contrast, knockdown of PG in LNCaP cells led to an increase in pSrc, demonstrating that PG inhibits Src activity in PCa cells (Fig. 5E).

### PG Alters ECM Molecular Profile in PCa Cells

The importance of changes in extracellular matrix (ECM) molecules for PCa growth, migration, invasion and metastasis has been well-established [44]–[47]. Recent evidence shows that PG is able to regulate ECM protein levels in keratinocytes [48]. In order to test whether PG regulates the levels of ECM molecules in PCa cell lines, we overexpressed PG or silenced PG expression in PCa cell lines and assessed the levels of vitronectin (VN), laminin (LN), fibronectin (FN), collagen I (Col I) and collagen IV (Col IV).

**Figure 6:**
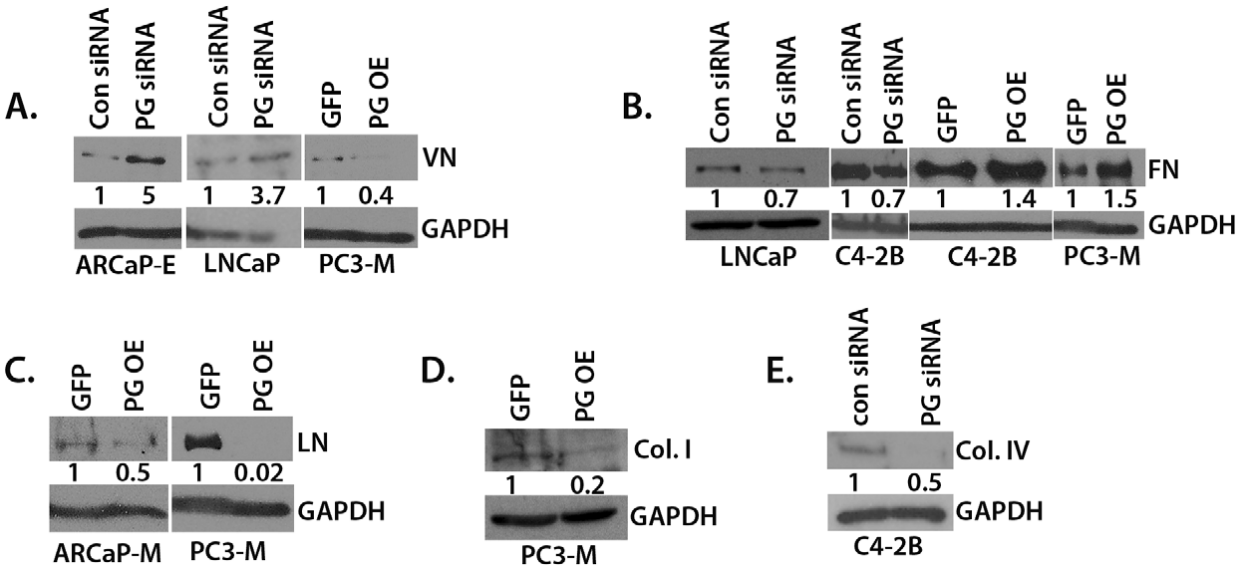
PG alters the profile of expression of ECM molecules in PCa cell lines. A–E. ARCaP_E_, ARCaP_M_, LNCaP, C4-2B, and PC3-M cells were plated in 6-well plates and allowed to attach and spread. The cells were either transduced with GFP- or PG-containing adenovirus and were lysed after 24 hours (PC3-M, C4-2B, ARCaP_M_); or the cells were transfected with control siRNA or PG siRNA pool and after 96 hours lysed (ARCaP_E_, LNCaP, C4-2B). The lysates were subjected to SDS-PAGE followed by immunoblotting with antibodies against VN (A), FN (B), LN (C), Col. I (D), Col IV (E) and GAPDH. Levels of ECM proteins were determined by normalizing to GAPDH levels for each cell line using Image J. Representatives of at least three independent immunoblots are shown, with numbers representing GAPDH normalized PG siRNA/Con siRNA or PG OE/GFP ratio for the blot shown. The average ratio and standard deviation of GAPDH normalized PG siRNA/Con siRNA and PG OE/GFP is as follows: (A) ARCaP_E_ 3.1+/−1.3, LNCaP 2.0+/−0.9, PC3-M 0.3+/−0.1; (B) LNCaP 0.7+/−0.05, C4-2B KD 0.8+/−0.1, C4-2B OE 1.2+/−0.2, PC3-M 1.5+/−0.05; (C) ARCaP_M_ 0.6+/−0.1, PC3-M 0.4+/−0.3; (D) 0.3+/−0.1; (E) 0.4+/−0.2.

Our results demonstrate that PG overexpression resulted in reduced VN, Col I and LN in PC3-M and ARCaP_M_ cells (Fig. 6A, C–D). Moreover, PG overexpression in PC3-M and C4-2B cells led to increased levels of FN. Using an siRNA pool to knockdown PG in ARCaP_E_, LNCaP and C4-2B cells resulted in reduced levels of FN and Col IV and an increase in VN (Fig. 6A–B, E). These data demonstrate the ability of PG to regulate ECM molecules in PCa cells.

### PG Inhibits Src Activation through Down-regulation of Vitronectin

**Figure 7:**
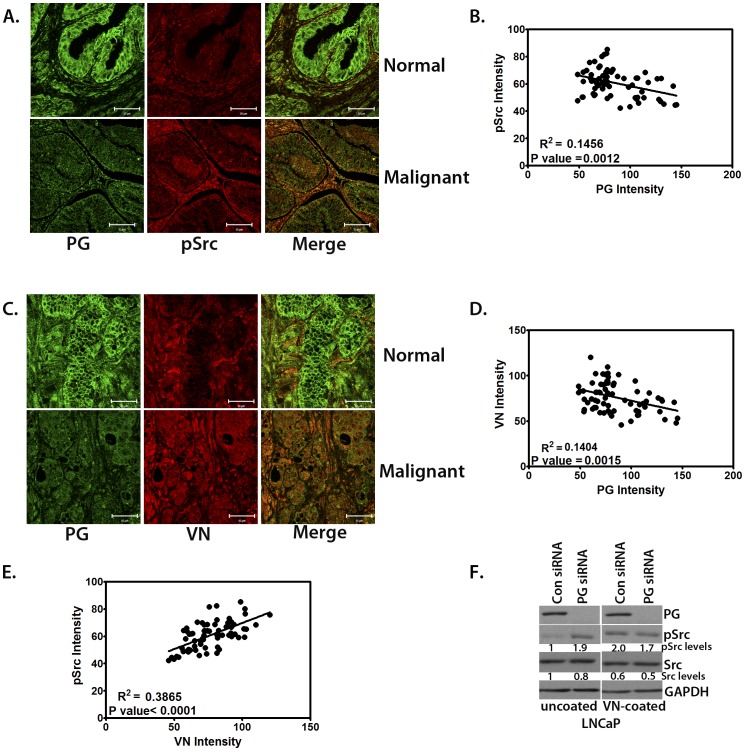
PG-dependent Src activity is regulated by VN. A. Confocal images showing PG and pSrc expression in normal and malignant prostate tissue (using 68 cores from the prostate TMA). PG is in green and pSrc is red. Bar, 50 µm. B. Scatterplot demonstrating the inverse correlation between pSrc expression and PG expression in prostate tissue (using 68 cores). C. Confocal images showing PG and VN expression in normal and malignant tissue (T2 samples were shown for both pSrc and VN as representative of average expression levels in all malignant tissue samples). PG is in green and VN is red. Bar, 50 µm. D. Scatterplot showing the inverse correlation between VN expression and PG expression in prostate tissue (using 68 cores). E. Scatterplot showing the direct correlation between VN expression and pSrc in prostate tissue (using 68 cores). F. Western blot showing PG, pSrc, and Src expression in LNCaP cells transfected with control or PG siRNA plated on uncoated or VN-coated plastic. VN rescues Src activation in the absence of PG.

We demonstrated that PG regulates PCa cell-cell adhesion and motility through inhibition of Src (Fig. 5). In addition, we found that PG is able to regulate ECM protein levels in PCa cell lines (Fig. 6). Since Src activity is known to be regulated through ECM interaction with integrins [49], we wanted to demonstrate the correlation between PG expression and ECM-dependent Src activation in tissue isolates from PCa patients. Therefore, we analyzed the levels of VN and activated Src in the tissue microarray. Out of the several ECM components analyzed, we chose to focus on VN, which, like Src, is instrumental for PCa bone metastasis through α_v_β_3_ integrin binding [21], [50]. In addition, VN is an extrinsic inducer of PCa tumor formation through regulation of cancer stem cell differentiation [51] making it an attractive candidate for regulation by PG. Moreover, side-by-side analysis of VN expression and Src activation in LNCaP cells shows a concomitant increase in VN levels and pSrc-Y416 in cells with suppressed PG compared to control (Fig. S4A). Using immunofluorescence staining for PG, pSrc-Y416, and VN in a prostate cancer tissue micro-array, we found that down-regulation of PG in tumor samples was usually followed by a sharp increase in abundance of VN and activated Src (Fig. 7A, C). Moreover, the scatterplots depicting the correlation between the relative fluorescence intensities of pSrc and VN in the prostate tissues as a function of PG expression levels demonstrated a clear increase in the levels of activated Src and VN in tumor tissues with decreased PG expression (Fig. 7B, D).

Interestingly, there was a strong positive correlation between Src activation and increased VN in PCa tissues (Fig. 7E). In order to test whether the underlying matrix regulated Src activity in PCa cells, we allowed ARCaP_E_ and ARCaP_M_ cells to deposit their own substrate and then removed cells by “de-roofing” while retaining basal cell components, including ECM [48]. Fresh ARCaP_E_ and ARCaP_M_ cells were then plated onto pre-deposited matrices and Src activity was analyzed. Plating ARCaP_E_ cells onto the VN-enriched ECM of ARCaP_M_ cells (Fig. S4B) caused an increase in pSrc-Y416, whereas plating ARCaP_M_ cells onto ARCaP_E_ ECM led to substantial down-regulation of p-Src-Y416 (Fig. S4C). Therefore, we decided to investigate the possibility that PG inhibits Src at least in part through down-regulation of VN. To achieve this, we plated control and PG-deficient LNCaP cells onto uncoated or VN-coated surfaces. We then lysed the cells and analyzed them by western blot for the presence of activated Src. On uncoated surfaces LNCaP cells had low levels of activated Src. However, in PG-deficient cells, Src was robustly activated (Fig. 7F). In contrast, plating LNCaP control cells on VN elicited strong activation of Src, similar to that observed in PG-deficient cells plated on an uncoated surface. When PG-deficient cells were placed on VN, they did not show any substantial additional Src activation, proving that the effect is not additive and likely mediated through the same pathway. These data suggest that PG-dependent Src activity is regulated by ECM composition, including VN.

## Discussion

The desmosomal armadillo protein plakoglobin is critical for cell-cell adhesion [52]. PG ablation results in severe loss of cell-cell adhesion strength [53] and of tissue integrity *in vivo* [54], [55]. In addition, in keratinocytes PG is involved in several cell-cell adhesion-independent activities, including cell motility [19], [20], Src and Rho activation [19], expression of cell-cell and cell-substrate adhesion components [48], transcription [56]–[58], and RNA stability [19]. However, despite being almost universally expressed in epithelial tissues [59], little is known of the function of PG outside its roles in skin and heart.

**Figure 8:**
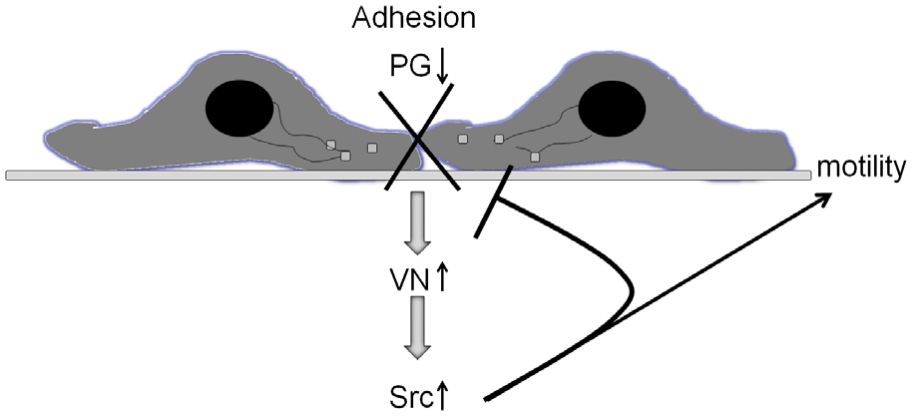
Model of the role of PG in prostate cancer adhesion and motility. Loss of PG results in a double negative hit on cell-cell junctions, while orchestrating alterations in extracellular matrix that promote motility. PG levels correspond directly to the strength of cell-cell adhesion in PCa cells. Down-regulation of PG leads to loss of cell-cell adhesion strength. Concomitantly, loss of PG causes up-regulation of VN and activation of Src. Together, these cell-cell and cell-ECM dependent effects of PG down-regulation lead to an increase in PCa cell motility.

Here we show that PG, a critical component of cell-cell adhesive junctions, regulates cell-substrate interactions, motility and invasion by suppressing vitronectin (VN)-dependent activation of Src. PG affects the levels of several ECM molecules, including down-regulation of vitronectin, which is instrumental in tissue invasion, metastasis, and tumor formation [21], [50], [51]. Interestingly, the down-regulation of PG in tumors coincides with an increase of both VN levels and Src activation in patient tissues, and our data demonstrate that exogenous VN is able to induce Src activation in cells where PG is suppressing Src activity. These findings are consistent with the idea that PG prevents tissue invasion of PCa through strengthening prostate tissue integrity, inhibiting motility and pro-invasive Src signaling through ECM remodeling and, specifically, down-regulating VN (Fig. 8). Overall, our data highlight the novel idea that desmosomal proteins regulate prostate cancer cell physical and signaling interactions with the underlying substrate.

It has been demonstrated that cell-cell and cell-ECM junctions can regulate each other’s function through controlling the expression, assembly and stability of their molecular components [60]–[64]. This regulation is especially critical during tumorigenesis where a complex pattern of physical and functional interactions emerges between cancer cells and their surrounding stroma [65]. In spite of these advances, very little is known about the junctional cross-talk in prostate cancer, especially involving desmosomal junction components. Despite their expression in prostate, the reports on the desmosomal molecules role in PCa progression have sometimes been conflicting [10]. The data presented here, building upon the previous functional studies in mouse keratinocytes [19], [20], lead us to hypothesize a new role for the desmosomal armadillo protein plakoglobin in PCa and epithelial tumorigenesis in general. Based on previous reports [53], [66] and our data presented here, we predict that PG helps maintain tissue integrity through strengthening cell-cell adhesions, regulating cell substrate production and interactions, and interfering with Src tyrosine kinase, thus preventing cells from leaving the epithelial sheet and invading into the surrounding stroma (Fig. 8). Its presence in a variety of epithelial tissues, including prostate, combined with its ability to regulate a number of ECM molecules, puts PG in the role of a sensor of the epithelial cell surroundings. In a normal epithelial sheet, most PG is localized in the cell-cell borders contributing to cell-cell adhesion strength and tissue cohesion. In a disrupted state, PG could relocate to the cytoplasm and nucleus where it would inhibit inappropriate responses by suppressing motility and invasion [19], [20], as well as promoting cell death [67]. This pathway would thus be down-regulated in all stages of epithelial cancers, including prostate. In early stages, down-regulation of PG may contribute to cell resistance to apoptosis, whereas in later stages it may facilitate invasion and metastasis. Alternatively, mutated forms of PG exhibiting altered adhesion-dependent and -independent functions could replace the wild-type molecule leading to similar effects [18]. Down-regulation or mutation of PG would allow for cancer cells to evade cell death and eventually leave their tissue of origin, invade into and survive in the surrounding stroma.

Apart from regulating cell-cell and cell-ECM adhesion and motility processes directly, PG has been shown to function as a modulator of several signaling molecules, including Src tyrosine kinase [19], [20]. Src kinase has been implicated in tumor progression of PCa, including promoting androgen-induced proliferation [68], expression of proangiogenic factors [69], motility [70], and bone metastasis of PCa cells [71]. Several lines of evidence implicate Src kinase in the invasiveness and metastatic progression of PCa [43], and its inhibitors are in clinical trials for the treatment of PCa [72]. It is believed that, in PCa, Src regulates cell adhesion, motility and invasion through activation of FAK and p130Cas [73]–[75]. It was recently demonstrated that PG suppresses keratinocyte motility in part through the inhibition of the Src/FAK signaling cascade [19]. Here we show the relevance of this inhibitory pathway in prostate cancer.

Invasive and metastatic abilities of PCa cells are controlled in large part by their interactions with and the remodeling of the surrounding matrix [44]. However, it is surprising to see that PG regulates the levels of ECM molecules in PCa cells, as cell-cell adhesion molecules have not previously been shown to regulate cell-ECM deposition and interactions in prostate cancer. The ability of PG to control the composition of the underlying matrix has been previously demonstrated in keratinocytes, where PG stabilizes FN mRNA thus leading to its overexpression [19]. Interestingly, we showed here that loss of PG led to substantial increase in VN, a matrix molecule whose presence is strongly implicated in PCa bone metastasis and tumor formation [21], [50], [51]. The apparent paradox of PG up-regulating FN in keratinocytes and down-regulating VN in PCa cells may lie in the separate mechanisms involved. In keratinocytes, FN mRNA was stabilized by the presence of PG. However, in the absence of PG, FN promoter activity increased over 10-fold [19]. PG has been established as a regulator of gene transcription, either directly or through regulating its closest relative β-catenin [76]. As PG is known to inhibit transcription in some systems [77] it is tempting to speculate that, in PCa, the presence of PG leads to inhibition of the VN promoter. One of the potential candidates for a mediator of PG suppression of VN is C/EBP, which has been shown to be regulated by PG [78]. Since the VN promoter contains C/EBP binding sites [79] it is possible that it could play a role in PG-mediated suppression of VN expression. As PG down-regulates VN in PCa, it follows that VN status could be directly correlated to PG’s inhibition of Src activity. We demonstrate here that VN is able to activate Src in cells with high PG expression to the levels achieved in PG deficient cells (Fig. 7). It would be interesting, however, to determine whether VN is the only molecule responsible for this PG activity, or if other PG-regulated ECM components are involved (Fig. 5).

In recent years strong evidence emerged connecting the loss of desmosomal adhesion with the conversion of *in situ* tumors to invasive cancers [80]. The data presented herein suggest a possible suppressive role for PG in PCa invasion and metastasis through cell-cell and cell-ECM adhesion inhibition of motility, in line with previous findings in prostate [8], [81], [82] and other cancers [83]–[85]. This makes PG an attractive molecule both as a potential diagnostic and therapeutic target in PCa. Therefore, further studies carefully dissecting distinct mechanisms of PG function in normal prostate and prostate cancer are warranted.

## Supporting Information

Figure S1 **Silencing and overexpression of PG in PCa cell lines.** A–E. Western blot demonstrating the overexpression or knockdown of PG in prostate cancer cell lines. ARCaP_E_ (A), LNCaP (C) or C4-2B (D) cells were transfected with control siRNA or PG siRNA pool, and 96 hours after transfection, the cells were lysed and subjected to SDS-PAGE, followed by immunoblotting with antibodies against PG and GAPDH. ARCaP_M_ (B) and PC3-M (E) cells were transduced with GFP-containing adenovirus or PG-containing adenovirus, and 24 hours later the cells were lysed and subjected to SDS-PAGE, followed by immunoblotting with antibodies against PG and GAPDH.
(TIF)

Figure S2 **Specific siRNA sequences used to ablate PG from PCa cell lines.** A. Western blot demonstrating the efficiency of 4 different individual siRNA sequences, as well as their mix, targeting PG in PC3-M cells. 20 nM final concentration of siRNA was used. B. Representative image of a dispase assay in PC3-M cells after transfection with the sequences shown in panel A. Representative of at least three independent immunoblots are shown, with numbers representing GAPDH normalized PG protein levels for the blot shown. The average level and standard deviation of PG protein levels in panel A is as follows: 0.4+/−0.04, 0.6+/−0.02, 0.3+/−0.1, 0.4+/−0.1, 0.6+/−0.2
(TIF)

Figure S3 **PG expression stabilizes adherens and desmosomal junction components in PCa.** A. Immunofluorescence staining showing the localization of E-cadherin in LNCaP cells after PG overexpression. B. Western blots showing expression of adherens and desmosomal junction components after overexpression or knockdown of PG in ARCaP_M_ and ARCaP_E_ cells, respectively.
(TIF)

Figure S4 **PG-dependent Src activity is regulated by ECM composition.** A. Western blot demonstrating increased levels of pSrc and VN after PG knock-down in LNCaP cells. B. Western blot showing an increase in VN in the more metastatic, low PG expressing ARCaP_M_ cells over less metastatic, high PG expressing ARCaP_E_ cells. C. Western blot representing an increase in pSrc levels in ARCaP_E_ cells plated onto the VN-rich matrix deposited by ARCaP_M_ cells (lanes 1 and 2) and a decrease in pSrc levels in ARCaP_M_ cells plated onto matrix deposited by ARCaP_E_ cells (lanes 3 and 4). Representatives of at least three independent immunoblots are shown, with numbers representing Src normalized pSrc levels for the blot shown in panel C. All the pSrc levels were normalized to the levels in ARCaP_E_ cells plated on ARCaP_E_ matrix within each blot. The average level and standard deviation of pSrc levels in panel C is as follows: E/M 2.6+/−0.9, M/M 4.8+/−1.1, M/E 1.2+/−0.5
(TIF)
