# Supplementary figures and images for: The Desmosomal Armadillo Protein Plakoglobin Regulates Prostate Cancer Cell Adhesion and Motility through Vitronectin-Dependent Src Signaling
Source: PLoS One. 2012 Jul 30;7(7):e42132. doi: 10.1371/journal.pone.0042132 (PMC3408445; doi:10.1371/journal.pone.0042132)

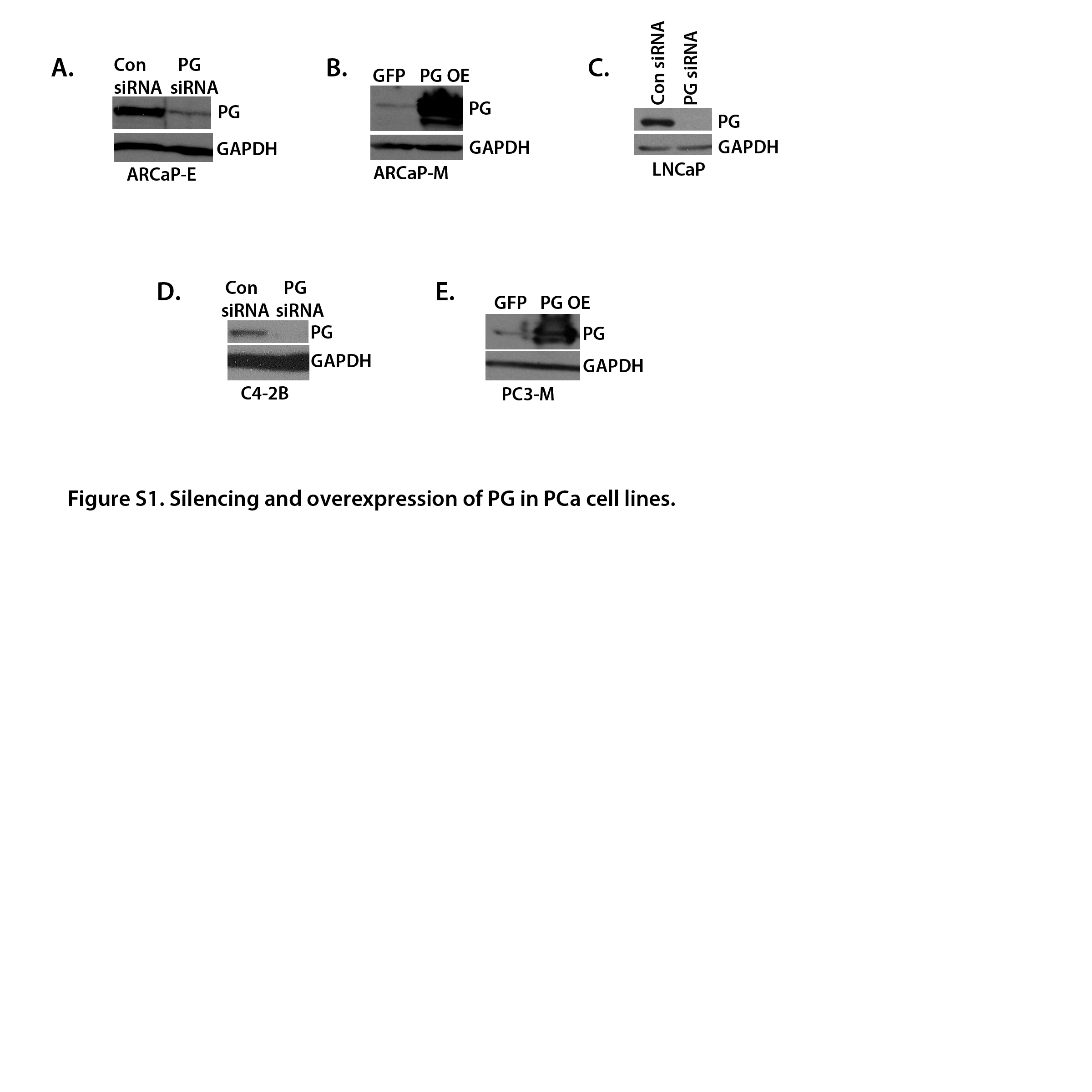

Supplement: Figure S1 — Silencing and overexpression of PG in PCa cell lines. A–E. Western blot demonstrating the overexpression or knockdown of PG in prostate cancer cell lines. ARCaPE (A), LNCaP (C) or C4-2B (D) cells were transfected with control siRNA or PG siRNA pool, and 96 hours after transfection, the cells were lysed and subjected to SDS-PAGE, followed by immunoblotting with antibodies against PG and GAPDH. ARCaPM (B) and PC3-M (E) cells were transduced with GFP-containing adenovirus or PG-containing adenovirus, and 24 hours later the cells were lysed and subjected to SDS-PAGE, followed by immunoblotting with antibodies against PG and GAPDH. (TIF) [file pone.0042132.s001.tif]

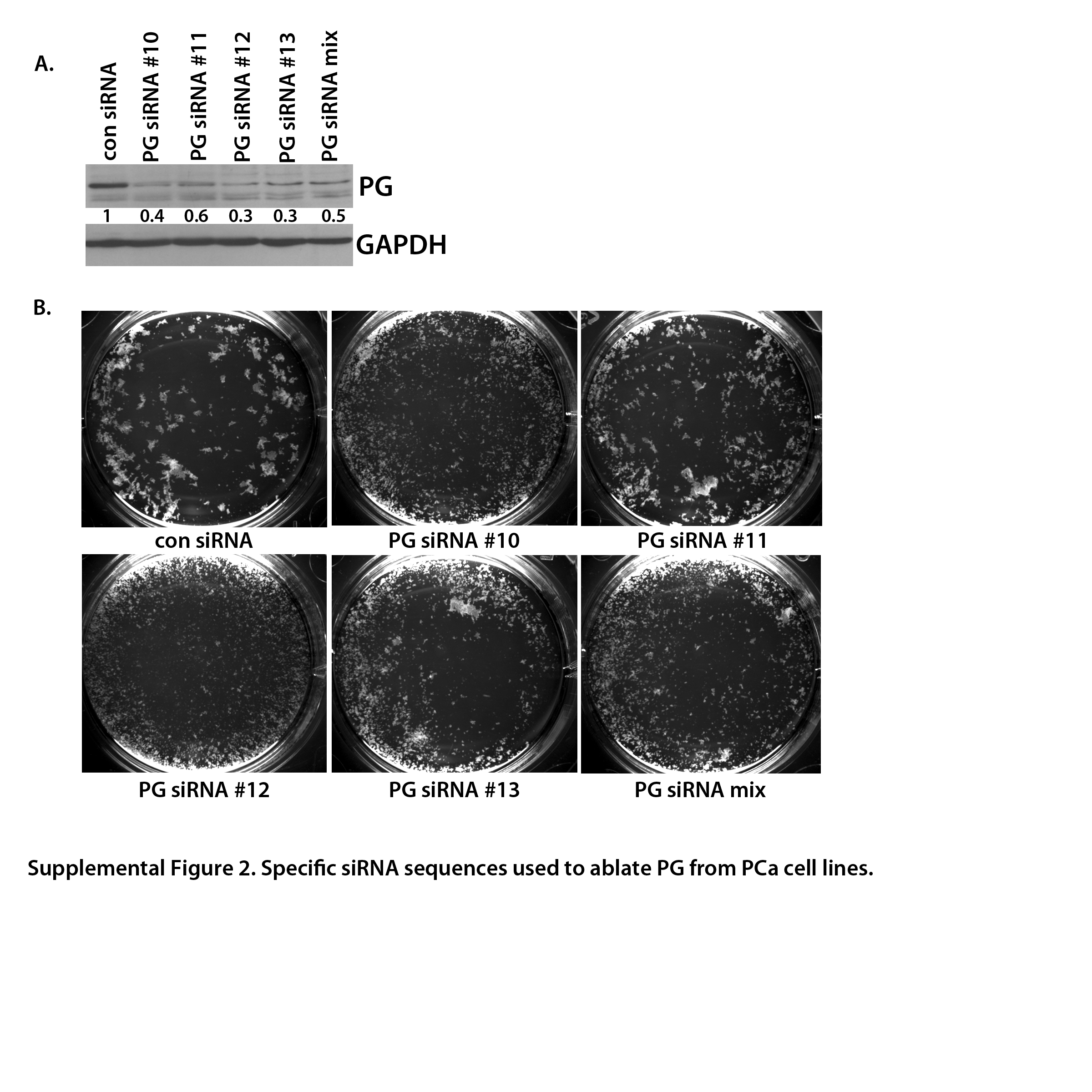

Supplement: Figure S2 — Specific siRNA sequences used to ablate PG from PCa cell lines. A. Western blot demonstrating the efficiency of 4 different individual siRNA sequences, as well as their mix, targeting PG in PC3-M cells. 20 nM final concentration of siRNA was used. B. Representative image of a dispase assay in PC3-M cells after transfection with the sequences shown in panel A. Representative of at least three independent immunoblots are shown, with numbers representing GAPDH normalized PG protein levels for the blot shown. The average level and standard deviation of PG protein levels in panel A is as follows: 0.4+/−0.04, 0.6+/−0.02, 0.3+/−0.1, 0.4+/−0.1, 0.6+/−0.2 (TIF) [file pone.0042132.s002.tif]

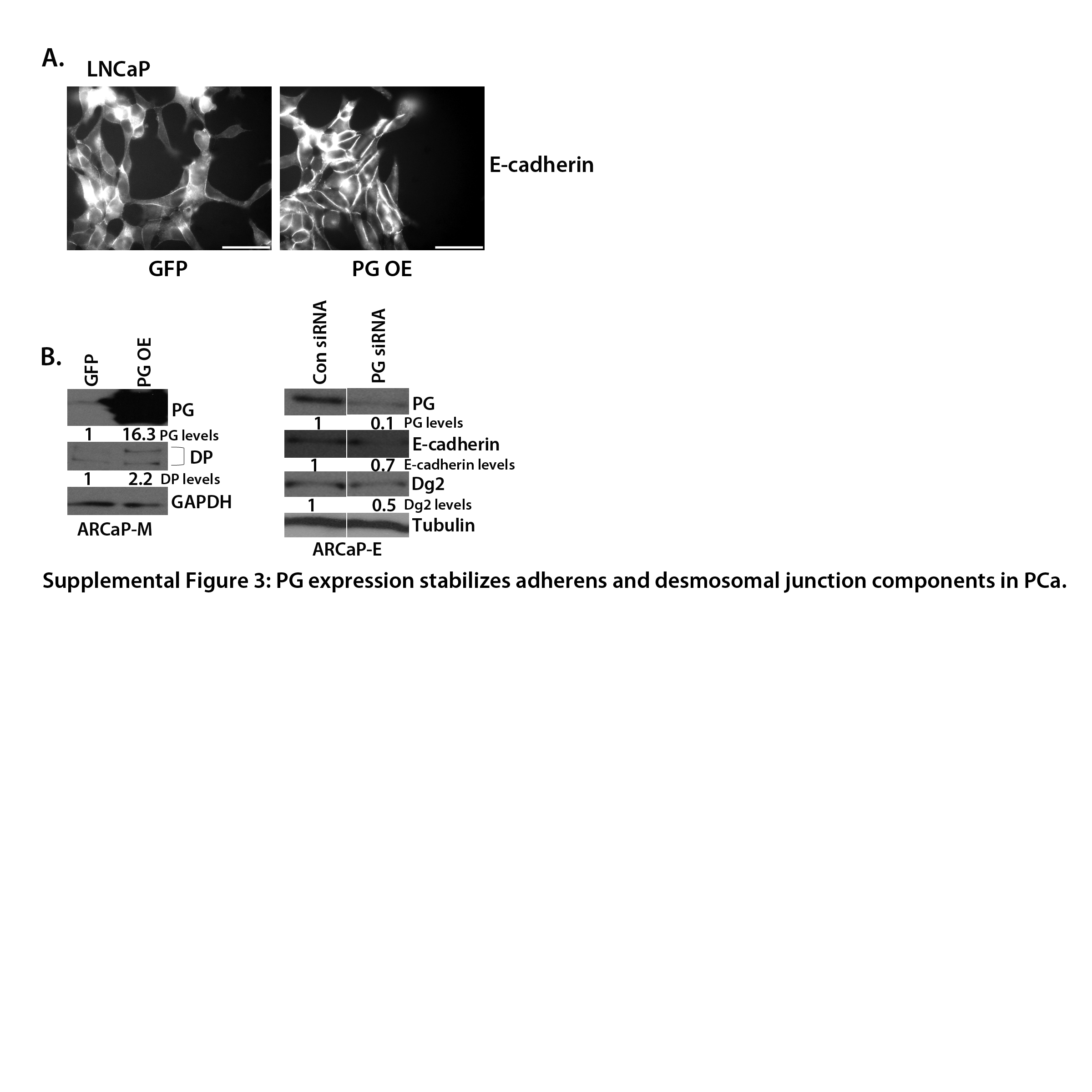

Supplement: Figure S3 — PG expression stabilizes adherens and desmosomal junction components in PCa. A. Immunofluorescence staining showing the localization of E-cadherin in LNCaP cells after PG overexpression. B. Western blots showing expression of adherens and desmosomal junction components after overexpression or knockdown of PG in ARCaPM and ARCaPE cells, respectively. (TIF) [file pone.0042132.s003.tif]

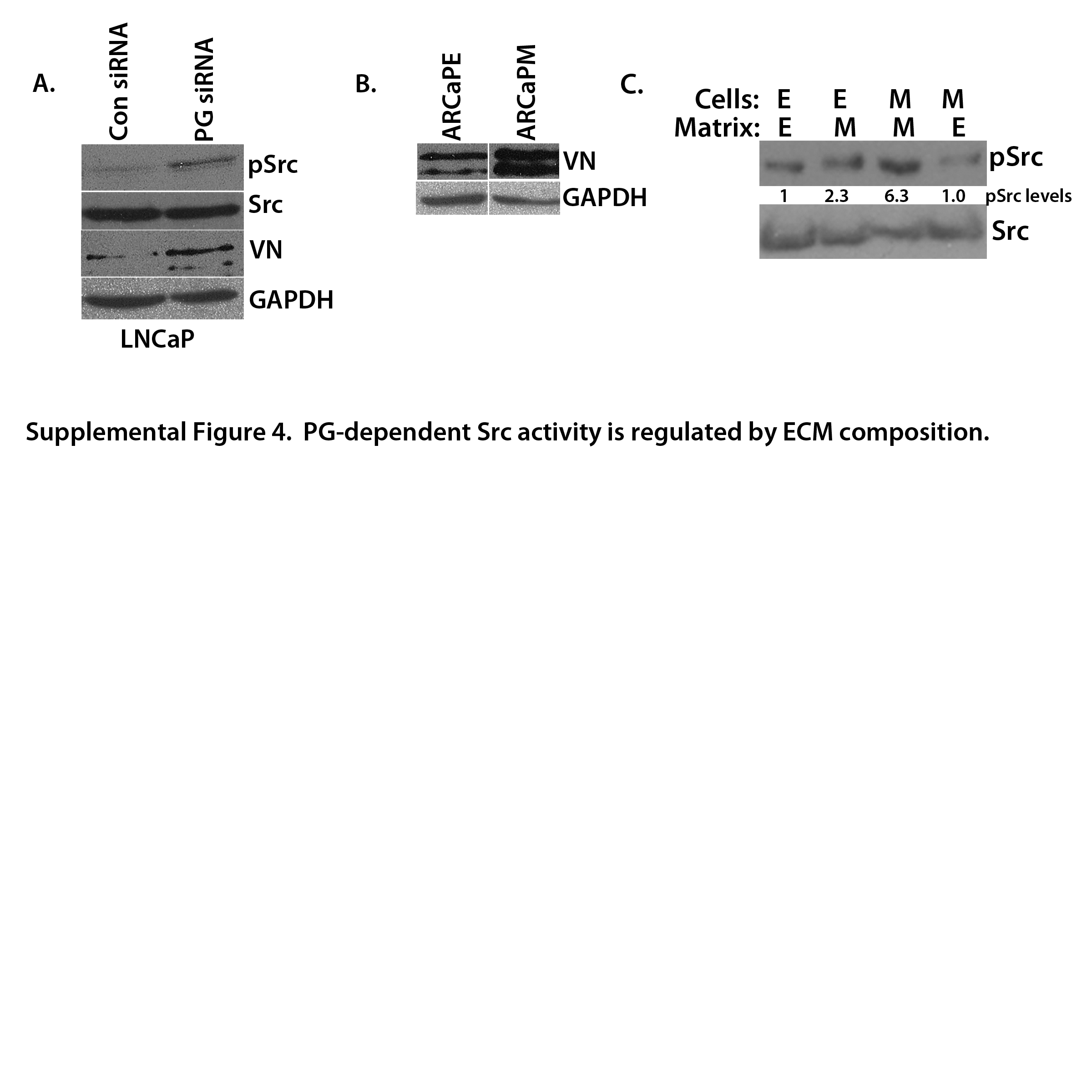

Supplement: Figure S4 — PG-dependent Src activity is regulated by ECM composition. A. Western blot demonstrating increased levels of pSrc and VN after PG knock-down in LNCaP cells. B. Western blot showing an increase in VN in the more metastatic, low PG expressing ARCaPM cells over less metastatic, high PG expressing ARCaPE cells. C. Western blot representing an increase in pSrc levels in ARCaPE cells plated onto the VN-rich matrix deposited by ARCaPM cells (lanes 1 and 2) and a decrease in pSrc levels in ARCaPM cells plated onto matrix deposited by ARCaPE cells (lanes 3 and 4). Representatives of at least three independent immunoblots are shown, with numbers representing Src normalized pSrc levels for the blot shown in panel C. All the pSrc levels were normalized to the levels in ARCaPE cells plated on ARCaPE matrix within each blot. The average level and standard deviation of pSrc levels in panel C is as follows: E/M 2.6+/−0.9, M/M 4.8+/−1.1, M/E 1.2+/−0.5 (TIF) [file pone.0042132.s004.tif]
